# Unsuspected osteochondroma-like outgrowths in the cranial base of Hereditary Multiple Exostoses patients and modeling and treatment with a BMP antagonist in mice

**DOI:** 10.1371/journal.pgen.1006742

**Published:** 2017-04-26

**Authors:** Sayantani Sinha, Christina Mundy, Till Bechtold, Federica Sgariglia, Mazen M. Ibrahim, Paul C. Billings, Kristen Carroll, Eiki Koyama, Kevin B. Jones, Maurizio Pacifici

**Affiliations:** 1 Translational Research Program in Pediatric Orthopaedics, Division of Orthopaedic Surgery, The Children’s Hospital of Philadelphia, Philadelphia, Pennsylvania, United States of America; 2 Shriner’s Hospital for Children, Salt Lake City, Utah, United States of America; 3 Department of Orthopaedics, University of Utah School of Medicine, Salt Lake City, Utah, United States of America; 4 Department of Oncological Sciences and Huntsman Cancer Institute, University of Utah School of Medicine, Salt Lake City, Utah, United States of America; Murdoch Childrens Research Institute, AUSTRALIA

## Abstract

Hereditary Multiple Exostoses (HME) is a rare pediatric disorder caused by loss-of-function mutations in the genes encoding the heparan sulfate (HS)-synthesizing enzymes EXT1 or EXT2. HME is characterized by formation of cartilaginous outgrowths—called osteochondromas- next to the growth plates of many axial and appendicular skeletal elements. Surprisingly, it is not known whether such tumors also form in endochondral elements of the craniofacial skeleton. Here, we carried out a retrospective analysis of cervical spine MRI and CT scans from 50 consecutive HME patients that included cranial skeletal images. Interestingly, nearly half of the patients displayed moderate defects or osteochondroma-like outgrowths in the cranial base and specifically in the clivus. In good correlation, osteochondromas developed in the cranial base of mutant *Ext1*^*f/f*^*;Col2-CreER* or *Ext1*^*f/f*^*;Aggrecan-CreER* mouse models of HME along the synchondrosis growth plates. Osteochondroma formation was preceded by phenotypic alteration of cells at the chondro-perichondrial boundary and was accompanied by ectopic expression of major cartilage matrix genes -*collagen 2* and *collagen X-* within the growing ectopic masses. Because chondrogenesis requires bone morphogenetic protein (BMP) signaling, we asked whether osteochondroma formation could be blocked by a BMP signaling antagonist. Systemic administration with LDN-193189 effectively inhibited osteochondroma growth in conditional *Ext1*-mutant mice. In vitro studies with mouse embryo chondrogenic cells clarified the mechanisms of LDN-193189 action that turned out to include decreases in canonical BMP signaling pSMAD1/5/8 effectors but interestingly, concurrent increases in such anti-chondrogenic mechanisms as pERK1/2 and *Chordin*, *Fgf9* and *Fgf18* expression. Our study is the first to reveal that the cranial base can be affected in patients with HME and that osteochondroma formation is amenable to therapeutic drug intervention.

## Author summary

The rare musculoskeletal pediatric disorder Hereditary Multiple Exostoses (HME) is characterized by benign cartilaginous outgrowths called osteochondromas that cause limb and axial skeletal problems and can also transform into life threatening chondrosarcomas. Surprisingly, no report has yet identified osteochondromas in the craniofacial skeleton though it contains several endochondral structures, including the cranial base. We report here for the first time that the cranial base of many HME patients does in fact exhibit defects and osteochondroma-like outgrowths and that similar lesions form in the cranial base of mouse models of HME. Of similar major importance is our novel demonstration that osteochondroma formation in mutant mice can be effectively inhibited by systemic treatment with a drug that blocks BMP signaling, an essential pro-chondrogenic pathway. Our study thus represents a major step ahead in understanding the pathogenesis and complexities of HME and provides proof-of-principle evidence that osteochondroma formation is amenable to drug-based treatment.

## Introduction

Hereditary Multiple Exostoses (HME, also known as Multiple Osteochondromas) is a rare autosomal-dominant pediatric disorder that affects about 1 in 50,000 individuals worldwide [1, 2]. HME is characterized by benign cartilage-capped outgrowths -referred to as osteochondromas or exostoses- that form in the perichondrium of growth plates in endochondral skeletal elements including long bones, vertebrae and ribs [3, 4]. Because of their location, size and number, osteochondromas can cause skeletal deformities, lengthening disparity, chronic pain, ligament and blood vessel or neurologic impingements, and early onset osteoarthritis [5]. Though the etiology of each of these problems is unclear, the skeletal deformities are likely caused by osteochondromas interfering with growth plate function and peripheral *vs* longitudinal bone growth [6]. As physical difficulties progress, patients undergo surgery to remove symptomatic osteochondromas and correct skeletal defects, with some patients undergoing numerous operations by age 18. In children, however, surgery can be dangerous as it can irreversibly damage the adjacent growth plate [5]. Although no new osteochondromas form after puberty when the growth plates close, existing osteochondromas can continue to cause complications eventually requiring surgery, leading to a surgical rate of over 65% in the HME patient population [7, 8]. Also, osteochondromas can transform into malignant chondrosarcomas in 1 to 2% of patients [9]. Except for surgery, there is no biological or pharmacological treatment available for HME at present.

The genes associated with most HME cases are *EXT1* and *EXT2* that encode components of a Golgi-resident co-polymerase complex responsible for heparan sulfate (HS) chain synthesis [10–12]. Most HME patients bear a germline heterozygous loss-of-function mutation in *EXT1* or *EXT2* and display a systemic HS decrease of about 50% [13]. A second hit may be needed to induce osteochondroma formation, and studies have reported loss-of-heterozygosity (LOH) or aneuploidy in some human osteochondromas rendering resident chondrocytes *EXT*-null [14, 15]. Other studies have described patients with alternative genetic changes including compound heterozygous *EXT1* and *EXT2* mutations [16–19]. In good correlation, we and others showed that while single heterozygous *Ext1*^*+/-*^ or *Ext2*^+/-^ mice were largely normal, compound heterozygous *Ext1*^*+/-*^*;Ext2*^*+/-*^ and conditional *Ext1*^*f/f*^*;Col2CreER*-null mice (both producing less HS than single heterozygous mice) displayed multiple osteochondromas and closely mimicked human HME [20–24]. Thus, these human and mouse studies have strongly indicated that osteochondroma formation requires a steep—albeit not necessarily complete- drop in HS levels.

The HS chains are constituents of cell surface syndecans and glypicans and other HS-rich proteoglycans that are essential for numerous physiologic processes, including skeletal growth [25–29]. They function most notably by interacting with key HS-binding signaling proteins -including bone morphogenetic proteins (BMPs) and hedgehog proteins- and restricting protein distribution, diffusion and action on target cells [25, 30, 31]. We previously showed there was broad, ectopic and excess BMP signaling in perichondrium of long bone growth plates in conditional *Ext1*-null mice that was followed by ectopic cartilage tissue formation and osteochondroma development [4, 27]. Because BMP signaling is a major pro-chondrogenic mechanism [32, 33], its ectopic action in perichondrium likely had a direct role in inducing ectopic cartilage and osteochondroma formation. Interestingly, the data also indicated that the BMP signaling pathway could represent a therapeutic target [4, 27].

The cranial base is a large endochondral skeletal structure with a complex morphology and origin on which the cerebrum, pituitary gland, pons and cerebellum lie [34, 35]. During embryogenesis and early childhood, the developing cranial base is characterized by the presence of dual mirror-image growth plates called synchondroses. The ethmoidal, intrasphenoidal and spheno-occipital synchrondroses are responsible for antero-posterior growth, elongation and lateral widening of the cranial base, respectively [36, 37]. The activity of each synchondrosis and the specific age at which each closes are intimately coordinated with overall growth and expansion of both the brain and the intramembranous skeletal elements of the cranial vault [38, 39]. Precocious closure or malfunction of the synchondroses can contribute to severe craniofacial skeletal abnormities such as those seen in pediatric disorders including Apert and Crouzon syndromes [40]. Despite their unique mirror-image arrangement, the synchondroses contain typical chondrocyte maturation zones and express and obey all the classic regulators of growth plate function seen in appendicular and axial skeletal elements, including BMP signaling [41–44]. They are also flanked by a perichondrium facing the brain and oral sides [44]. Therefore, the synchondroses and cranial base should theoretically be affected by the *EXT* mutations and HS deficiency that alter axial and appendicular elements in HME. However, we know of no previous study that analyzed the cranial base of HME patients for the presence of defects and/or osteochondromas. Given the obvious importance of this question and possible medical ramifications, we have retrospectively examined the MRI and CT scans of 50 consecutive HME patients seen at one of our hospitals. We also asked whether conditional *Ext1* ablation would alter the cranial base and cause osteochondroma formation in mice and whether osteochondroma formation was amenable to drug treatment. The present study provides novel data and affirmative answers to all these critical questions.

## Results

### HME patients display cranial base irregularities and osteochondroma-like outgrowths

**Fig 1:**
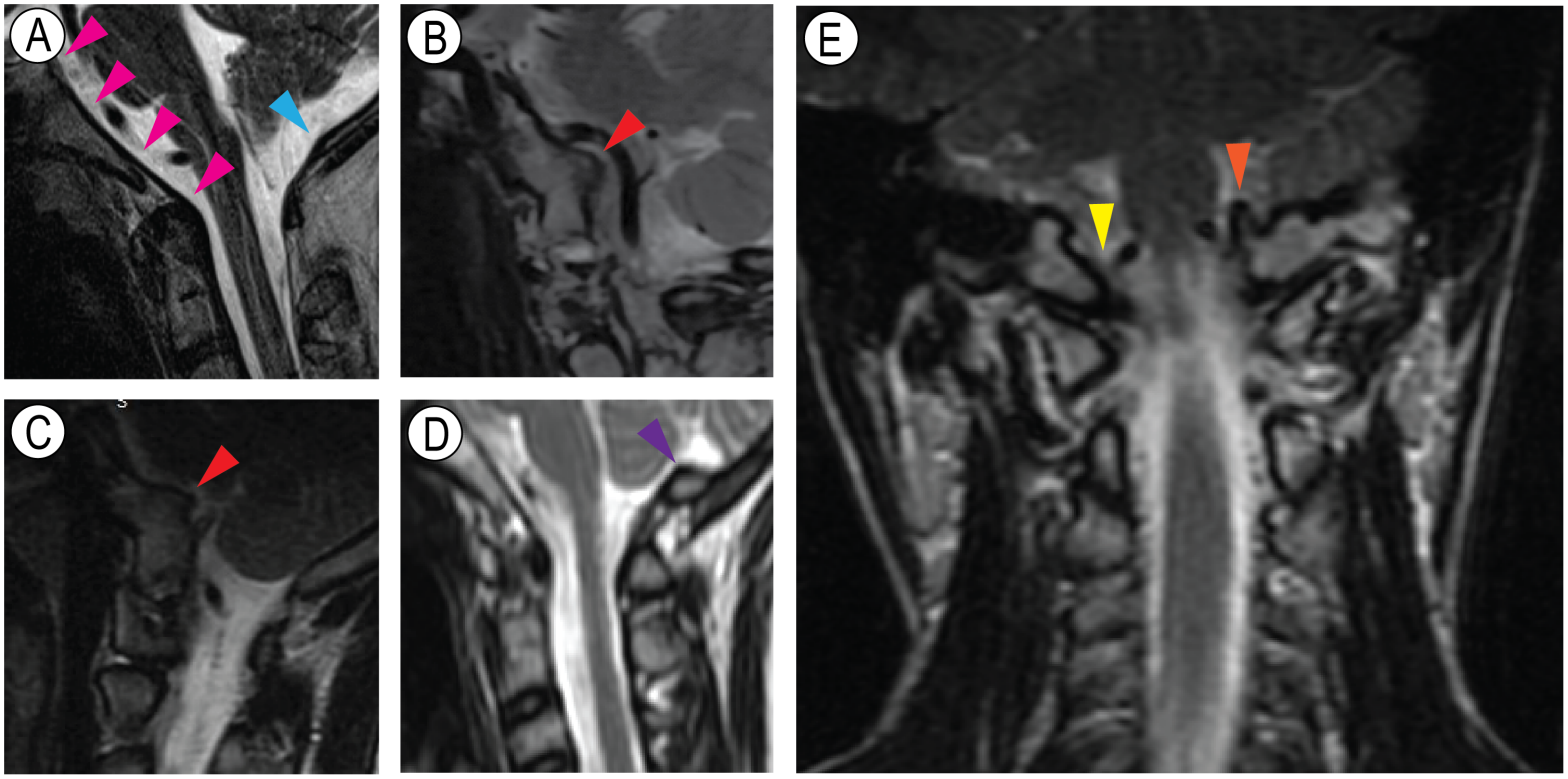
HME patients have cranial base defects. (**A**) Sagittal plane T2 MRI sequence showing an example of a normal clivus (delineated by pink arrowheads) and normal posterior foramen magnum (cyan arrowhead) in a HME patient. (**B-C**) Sagittal plane T1 and T2 MRI sequences showing a very large osteochondroma-like outgrowth (red arrowheads) in a patient that is characterized by continuity with clivus cortical bone and marrow. For orientation, note that the cerebellum is the large tissue mass occupying the upper right half of the images. (**D**) Sagittal T2 MRI sequence from a patient displaying an irregularity in the occipital lip of the foramen magnum (purple arrowhead). (**E**) Coronal plane T2 MRI sequence showing an osteochondroma-like outgrowth (orange arrowhead) and a more normal contralateral side (yellow arrowhead).

Osteochondromas develop in many axial and appendicular skeletal elements in HME patients and can vary in number and size from patient to patient [5, 45]. The osteochondromas in the spine can emerge on the outer perimeter of the vertebrae, but can also develop inside the spinal canal and impinge on the spinal cord [46]. In response to evidence that such intra-spinal osteochondromas are prevalent and can be particularly dangerous, a protocol was developed according to which patients diagnosed with HME are routinely subjected to total spine imaging by MRI or CT scan at one of our hospitals [47]. This provided us with an opportunity to carry out a retrospective examination of the scans of 50 consecutive HME patients. Because the scans were ordered for the spine, they did not include the entire skull and contained varying portions of cephalad extent of cervical spine and skull structures. Nonetheless, the scans consistently included the posterior cranial base, clivus, cervical vertebrae, foramen magnum and portions of the occiput flanked by surrounding tissues and organs on both the cranial and nasal/oral sides, including the cerebellum (Fig 1). In total, we reviewed 48 MRI scans and 2 CT scans of the 50 consecutive patients. Quite interestingly, the scans provided evidence for the occurrence of cranial base defects in a large proportion of patients (Table 1). In about 48% of the patients, the irregularities were very obvious and included a prominent and large outgrowth directed from the clivus toward surrounding tissues and structures including neighboring large vessels (Fig 1B and 1C, arrowheads). Such outgrowths showed both medullary and cortical bone continuity with the clivus itself and thus, displayed a most typical and diagnostic trait of osteochondromas [48]. Approximately 12% of the patients exhibited irregularities and bone asymmetries in the region (Fig 1D and 1E, arrowheads) that were readily appreciable when compared to patients without changes and displaying smooth and continuous bone contours (Fig 1A, arrowheads). The CT scans from two patients did not reveal changes. We should point out that none of the patients had neurological symptoms or findings on examination reported in their clinical notes.

**Table 1:** Base of skull imaging findings in HME patients.

Status	Number	Percentage	M:F gender ratio	Age (years)^a^
Normal	20	40	1:1	13.8 ± 3.8
Irregularity^b^	6	12	1:1	12.5 ± 4.2
Osteochondroma(s)	24	48	2:1	11.7 ± 3.5

^a^Mean ± standard deviation. The normal and osteochondroma group mean ages had a 2-tailed t-test p-value of 0.07.

^b^Some bone asymmetry or irregularity were identified, but there were no clear osteochondroma-like outgrowths with medullary and cortical bone continuity that are features necessary for the radiographic definition of an osteochondroma.

### Cranial base defects occur in mouse models of HME

**Fig 2:**
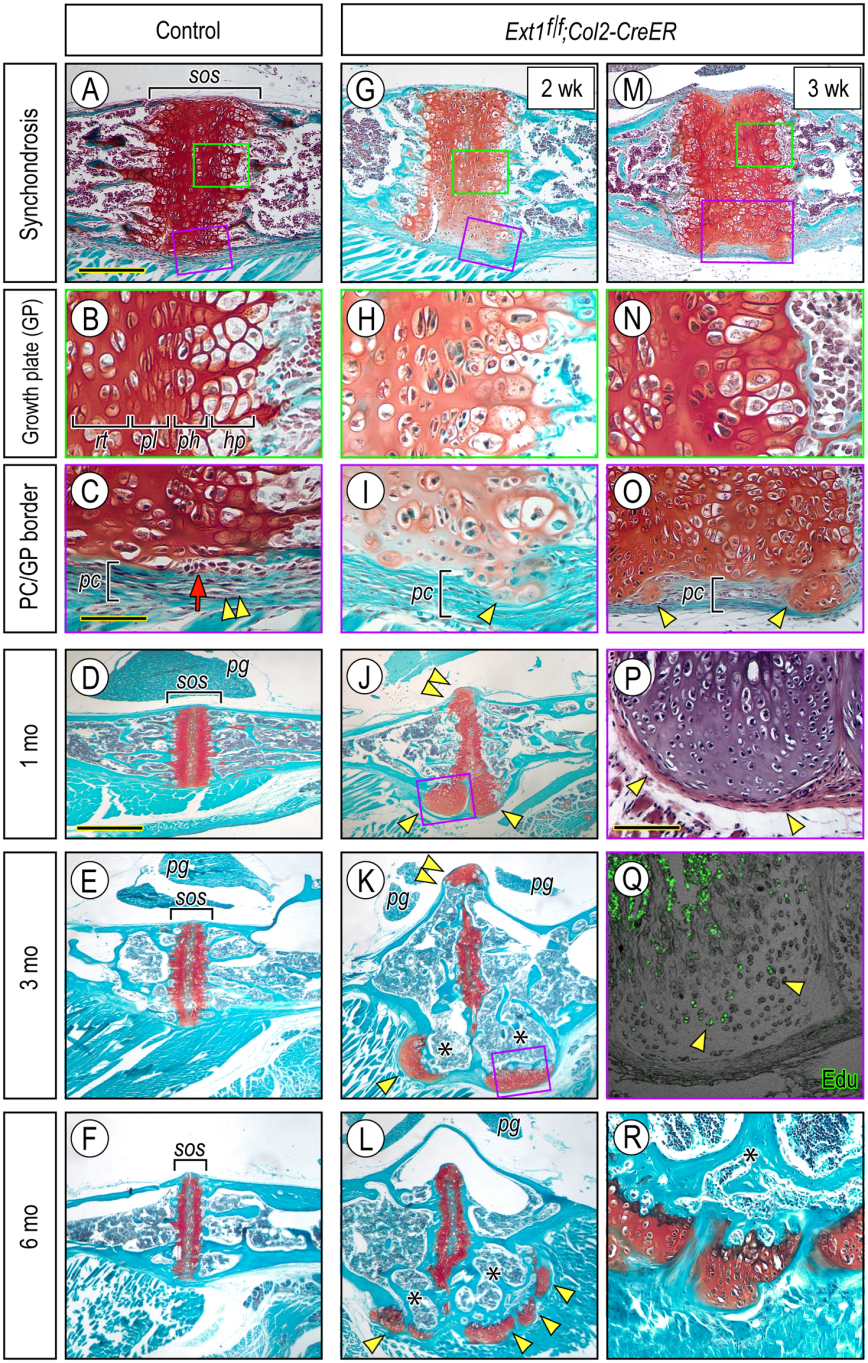
Osteochondromas form in the cranial base of *Ext1* mutant mice over time. (**A-F**) Longitudinal sections through the spheno-occipital synchondrosis (*sos*) in control tamoxifen-injected mice at 2 weeks (A-C), 1 month (D), 3 months (E) and 6 months (F) from injection stained with safranin O and fast green. Note in (B) the characteristic growth plate organization with resting (*rt*), proliferative (*pl*), prehypertrophic (*ph*) and hypertrophic (*hp*) zones, and note in (C) the distinct inner and outer portions of perichondrium (*pc*) (arrow and double arrowhead, respectively) along the perichondrium/growth plate (PC/GP) border. (**G-R**) Longitudinal sections through the spheno-occipital synchondrosis in *Ext1*^*f/f*^*;Col2-CreER* mutant mice at 2 weeks (G-I), 3 weeks (M-O), 1 month (J, P), 3 months (K, Q) and 6 months (L, R) from tamoxifen injection. Note that the chondro-perichondrial border is already deranged at 2 weeks as indicated by presence of round, safranin O-positive cells within perichondrium (I, arrowhead). By 3 weeks and thereafter, the border becomes occupied by incipient osteochondromas (O, arrowheads) that enlarge (J) and ossify proximally over time (K and L, asterisks) and maintain a characteristic cartilage cap at their distal end (K and L, arrowheads) with a growth plate-like organization (P and R) with scattered Edu-labeled proliferative cells (Q, arrrowheads). Bar in (A) for A, G and M, 1 mm; bar in (C) for B, C, H, I, N and O, 50 μm; bar in (D) for D, E, F, J-L and R, 3 mm; bar in (P) for P and Q, 300 μm.

**Fig 3:**
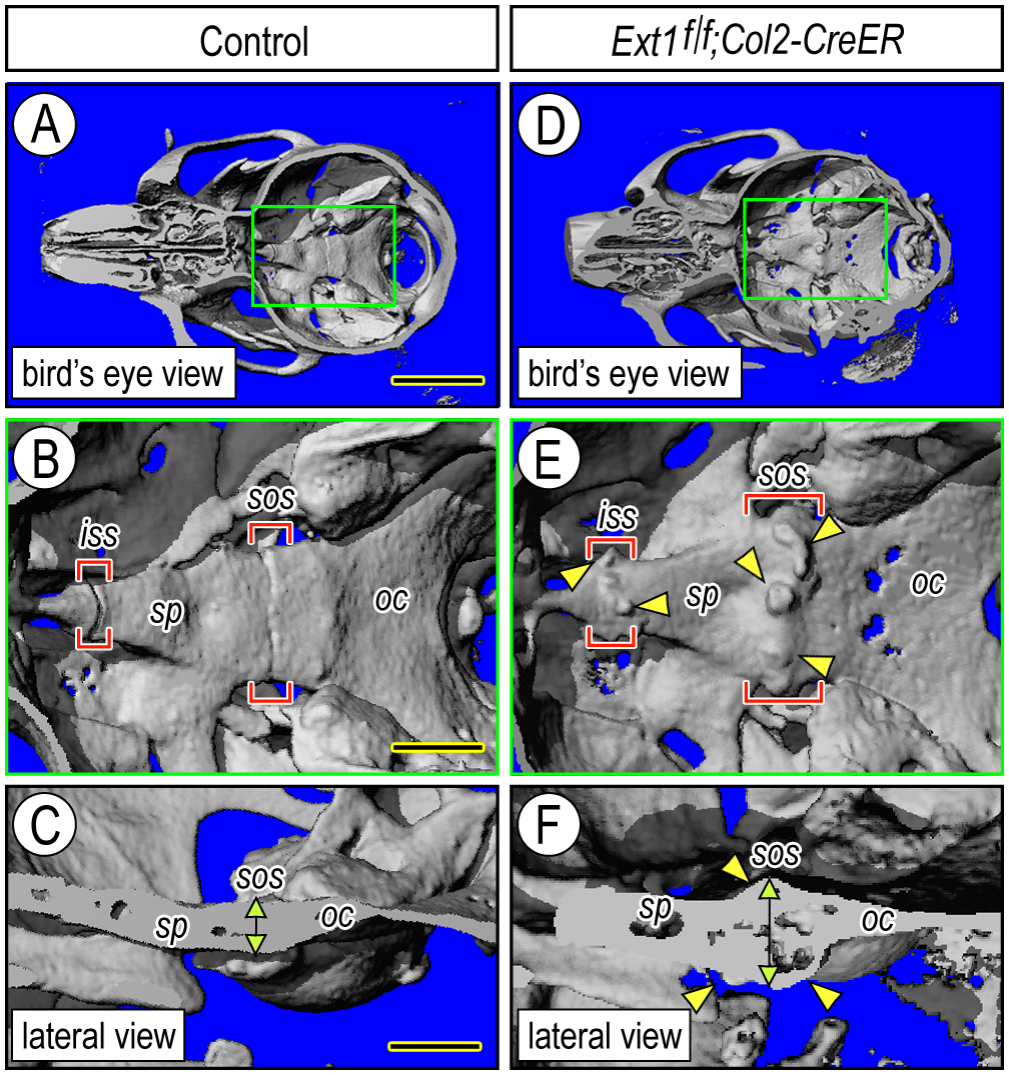
Imaging reveals multiple cranial base defects in mutant mice. (**A-B**) Bird’s eye μCT images of the skull from control tamoxifen-injected mice at 5 month time point from nasal end (left) to occipital area (right). At higher magnification (B), square area in green shows the smooth and continuous contour of the intrasphenoidal (*iss*) and spheno-occipital (*sos*) synchondroses flanked by the sphenoidal (*sp*) and occipital (*oc*) bones. (**C**) Lateral μCT image showing that the control cranial base is flat and relatively thin along and around the synchondroses (vertical arrow) as expected. (**D-F**) Bird’s eye and lateral μCT images of the skull from companion mutant tamoxifen-injected *Ext1*^*f/f*^*;Col2-CreER* mice showing that multiple osteochondromas (E, arrowheads) developed near the *iss* and *sos* synchondroses (bracketed areas in E) and that the mutant cranial base was considerably and abnormally thickened (vertical arrow) compared to controls (C). Bar in (A) for A and D, 5 mm; bar in (B) for B and E, 1.3 mm; and bar in (C) for C and F, 1 mm.

The above findings raised the question as to whether mouse models of HME would display comparable cranial base defects. To address this important question, we resorted to genetic approaches similar to those described in previous studies [20, 22, 49], using floxed *Ext1* mice mated with transgenic *Col2-CreER* mice that target growth plate chondrocytes and flanking perichondrial cells [50, 51]. Thus, we generated *Ext1*^*f/f*^*;Col2-CreER* pups, injected them with tamoxifen once at postnatal day 7 (P7) or P10, and then monitored and examined them over time post-injection. Controls consisted of companion *Ext1*^*f/+*^*;Col2-CreER* or *Ext1*^*f/f*^ mice injected with tamoxifen or vehicle. Mice were sacrificed at 1, 2, 3, 4 and 8 weeks and 3 and 5 months from the time of injection to capture and analyze the onset, progression and evolution of possible cranial base defects; rib and long bone samples were harvested from the same animals to contrast the cranial base with typically-affected axial and appendicular skeletal elements. As shown in Figs 2 and 3, mutant mice did develop cranial base defects and typical osteochondromas over time. In controls and at any time examined, the cranial base displayed a typical organization that included synchondroses, such as the spheno-occipital synchondrosis (*sos*), with their dual mirror-image growth plates and characteristic zones of resting, proliferating and hypertrophic chondrocytes all positive for Safranin O staining (Fig 2A and 2B). The synchondrosis border on the cranial and nasal sides displayed a thick, flat and continuous perichondrium that contained typical small oval and elongated progenitors in its inner portion (Fig 2C, arrow) and a fibrous compact layer in its outer half (Fig 2C, double arrowhead). In mutants at 2 and 3 weeks post-tamoxifen injection, the perichondrial contour was already distorted and uneven (Fig 2G, 2I, 2M and 2O), and round and enlarged cells occupied its inner half and protruded into the outer half (Fig 2I and 2O, arrowheads). Such round cells were likely to represent the initial responders to *Ext1* ablation, and their architecture and positive staining with Safranin O revealed their neo-chondrogenic character (Fig 2I and 2O, arrowheads). Given the well-known roles of *Ext1* and heparan sulfate in growth plate organization and function [27, 28, 52], it was not surprising to find that the synchondroses themselves became disorganized in mutants over time, and the zones of chondrocyte maturation progressively lost their sharp delineation and cellular characteristics (Fig 2H and 2N) as compared to normal zones in controls (Fig 2B).

By 4 weeks after tamoxifen injection, there were large cartilaginous outgrowths protruding away from the synchondrosis surface into the nasal and cranial sides (Fig 2J, arrowheads and double arrowhead, respectively) and displaying a typical growth plate-like organization (Fig 2P) and a thick perichondrium (Fig 2P, arrowheads). By 3 and 6 months from tamoxifen injection, the proximal portions of the osteochondromas had ossified and maintained medullary and cortical continuity with host bone (Fig 2K and 2L, asterisks) as previously seen in mutant long bones [22], and their distal ends displayed a characteristic cartilage cap (Fig 2K and 2L, arrowheads) with a growth plate-like organization (Fig 2Q and 2R) and scattered Edu-labeled proliferative cells (Fig 2Q, arrowheads). Outgrowths were absent in companion controls at any time point analyzed (Fig 2D–2F). Analysis by micro-computed tomography (μCT) of skulls at 5 to 6 month time points confirmed that the ossified osteochondromas protruded away from the cranial base surface (Fig 3D and 3E, arrowheads) toward the brain and nasal cavities at the sites of both the intrasphenoidal (*iss*) and spheno-occipital (*sos*) synchondroses (Fig 3D and 3E, brackets), while those sites were smooth and continuous in companion controls (Fig 3A and 3B). Lateral views of the μCT scans revealed that the cranial base had thickened considerably at the location of the osteochondromas (Fig 3F, arrowheads and brackets) compared to controls (Fig 3C). The mutant mice also displayed skeletal defects and large osteochondromas in ribs and long bones as seen previously [22].

**Fig 4:**
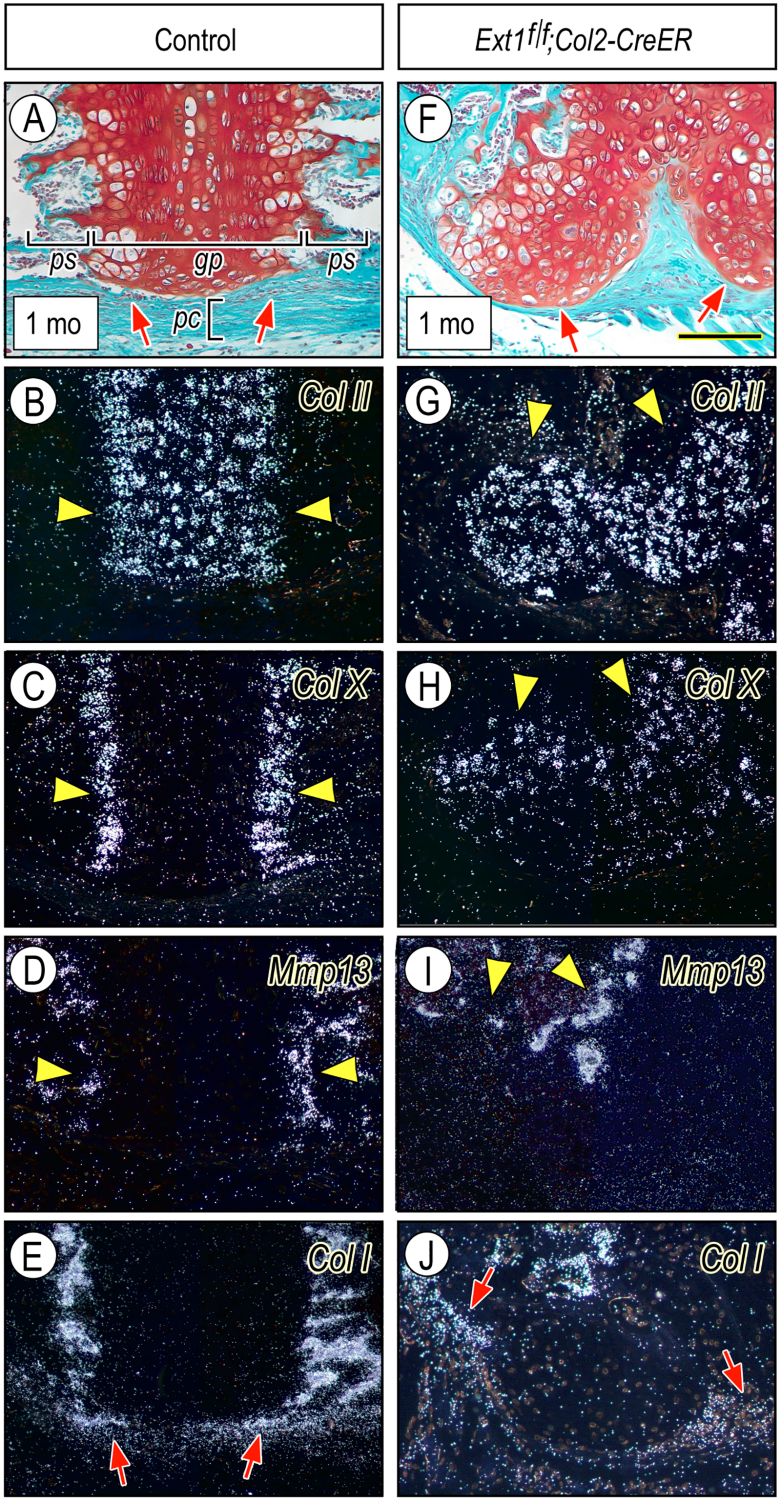
Cranial base osteochondromas display growth plate-like gene expression patterns. (**A-E**) Longitudinal serial sections through the spheno-occipital synchondrosis and flanking tissues from control mice at one month from tamoxifen injection. Sections were stained with safranin O-fast green to reveal cartilage organization and intact perichondrium (*pc*) and border (arrows) (A), and were processed for in situ hybridization expression analysis of such typical growth plate genes as: (B) collagen II (*Col II*); (C) collagen X (*Col X*); (D) metalloprotease 13 (*Mmp13*); and (E) collagen I (*Col I*). Arrowheads in B-D point to characteristic areas/sites of maximal gene expression in growth plates and arrows in E point to *Col I* expression in bone collar. (**F-J**) Longitudinal serial sections through osteochondromas present along the nasal aspect of cranial base in companion mutant tamoxifen-injected *Ext1*^*f/f*^*;Col2-CreER* mice. Arrows in F point to osteochondromas protruding away from the cranial base into surrounding perichondrium and nasal cavity. Arrowheads in G-I point to areas of maximal and somewhat deranged gene expression patterns within the osteochondromas. Arrows in J point to *Col I* expression in bone collar. Bar in (F) for all panels, 200 μm.

To more accurately define the character and phenotype of the affected cells, we carried out gene expression analyses by in situ hybridization. In controls, the synchondrosis growth plates expressed typical gene markers [41, 44] that included: collagen II (*Col II*) in top zones; collagen X (*Col X*) in the hypertrophic zones; metalloprotease 13 (*Mmp13*) in the mineralizing zones (Fig 4A–4D, arrowheads); and collagen I (*Col I*) in underlying primary spongiosa (Fig 4E). There was also characteristic and restricted *Col I* expression in the intramembranous bone collar forming in periosteum flanking the hypertrophic zones (Fig 4A and 4E, arrows). In companion mutants at 4 weeks post-injection, the enlarging and protruding osteochondromas displayed strong *Col II* expression in their more distal portion (Fig 4F and 4G) and *Col X* expression more proximally (Fig 4H, arrowheads). Reflecting the deranged growth plate-like organization within the osteochondromas, the expression patterns of *Mmp13* and *Col I* were scattered (Fig 4I and 4J, arrowheads and arrows, respectively), and low *Col I* expression characterized the entire distal fibroblastic contour of the osteochondromas (Fig 4J) with stronger expression in flanking bone collar (Fig 4J, arrows).

**Fig 5:**
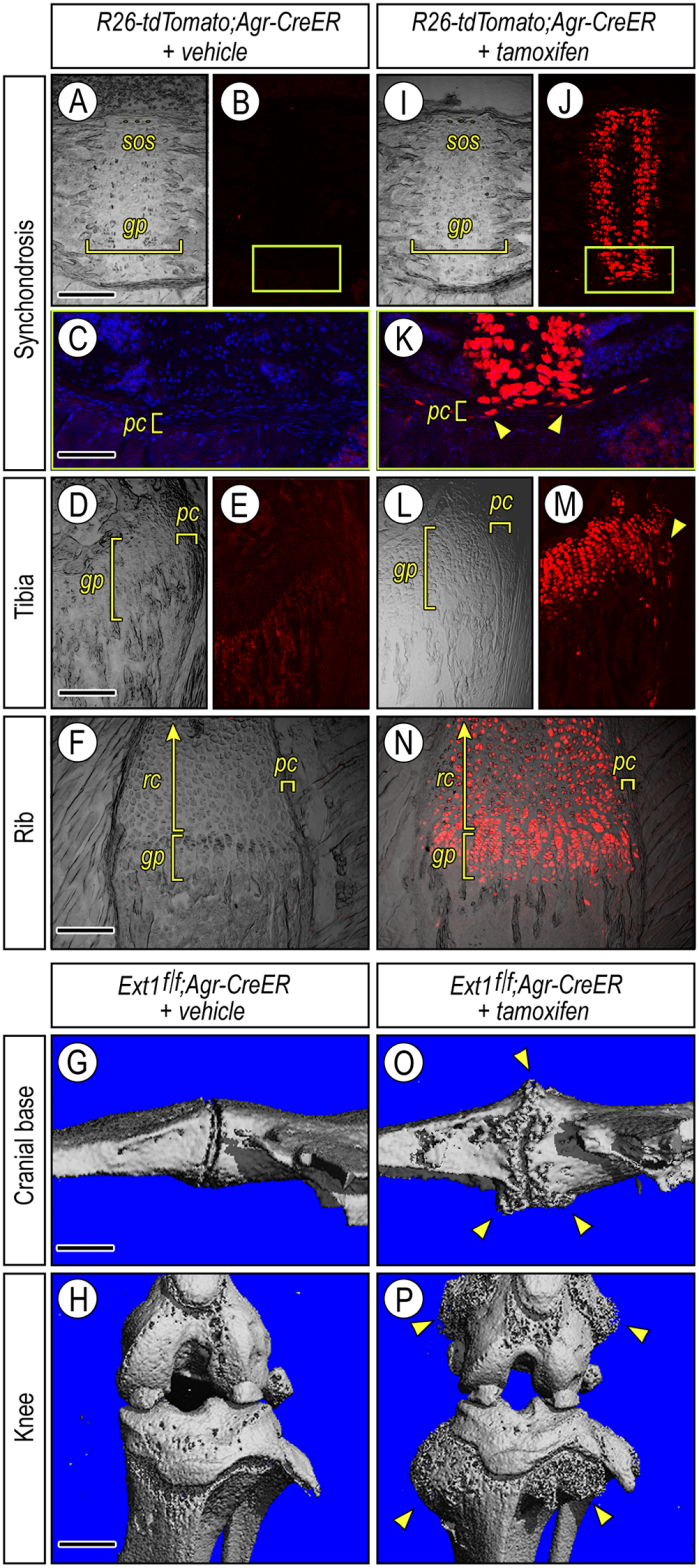
Stereotypic osteochondromas form in a juvenile HME mouse model. (**A-F**) Bright field and fluorescence images of longitudinal sections of spheno-occipital (*sos*) synchondroses (A-C), tibia (D-E) and ribs (F) from 5 week-old control *R26-tdTomato;Agr-CreER* mice that had been injected with vehicle 3 days earlier. The fluorescence images (B and E) show absence of reporter activity in growth plates (*gp*) and perichondrium (*pc*) at any anatomical site examined and thus, lack of *CreER* leakage in this model. Note that ribs contain a large resting cartilage (*rc*) adjacent to the growth plate (F). (**G-H**) μCT images of cranial base and knee from control *Ext1*^*f/f*^*;Agr-CreER* mice sacrificed 6 weeks from vehicle injection and displaying normal and typical anatomical characteristics. (**I-N**) Bright field and fluorescence images from companion *R26-tdTomato;Agr-CreER* mice that were administered tamoxifen once and were sacrificed 3 days post-injection. Note that reporter activity is very strong in spheno-occipital synchondrosis (*sos*), tibia and rib growth plates and rib resting cartilage (J, K, M and N) and is also conspicuous in flanking perichondrial (*pc*) cells at each anatomical location (arrowheads in K and M). Boxed area in J is shown at higher magnification in K. (**O-P**) μCT images of cranial base and knee from mutant *Ext1*^*f/f*^*;Agr-CreER* mice sacrificed 6 weeks after tamoxifen injection. Note the large multiple osteochondromas (arrowheads) protruding away from the bone surfaces at each anatomical site and better appreciable when contrasted to corresponding images from companion controls (G-H). Bar in (A) for A, B, I and J: 300 μm; bar in (C for C and K, 150 μm; bar in (D) for D, E, L and M, 250 μm; bar in (F) for F and N, 150 μm; and bar in (G) for G and O, 0.8 mm; and in (H) for H and P, 5 mm.

The *Col2-CreER* mouse line used above remains popular in HME research [20, 22, 49] and it was thus important for us to use it first here, but a major limitation of that line is that its transgene expression begins to drop around P7 and essentially ceases around P14 [50]. Thus, that line did not allow us to examine osteochondroma initiation and growth at later postnatal stages and in particular juvenile stages of rapid skeletal growth that are thought to be most conducive to osteochondroma formation and accumulation in patients [2, 45]. Thus, we carried out additional experiments using the transgenic *Aggrecan-CreERT2* (*Agr-CreER*) mouse line in which transgene expression persists through postnatal life [53]. To make sure this line elicited recombination in the cranial base, we produced *R26-tdTomato;Agr-CreER* reporter mice, injected them with tamoxifen once at juvenile stages (P21, P28 or P35) and assessed extent and topography of reporter expression over the following few days. Already by day 2 or 3 after tamoxifen injection, reporter activity was strongly activated and detectable through the bulk of synchodrosis growth plates (Fig 5I and 5J) as well as in cells located along the chondro-perichondrial border (Fig 5K, arrowheads), thus mimicking patterns elicited by *Col2-CreER* mice [51]. Virtually identical patterns of reporter topography were seen in long bones and ribs of the same animals (Fig 5L–5N). There was no detectable reporter activity in companion *R26-tdTomato;Agr-CreER* mice that were injected with vehicle (Fig 5A to 5F) nor was activity detected when these mice were examined at later time points as originally shown by Henry el., 2009 [53], verifying that nuclear entry of Cre-recombinase and recombination activity of this line are not leaky in the absence of tamoxifen. Thus, *Ext1*^*f/f*^*;Agr-CreER* mice were injected with tamoxifen once at P28 or P35, and companions were injected with vehicle as controls. By 6 to 8 weeks after injection, large osteochondromas had formed along the synchondroses in tamoxifen-treated mice and displayed characteristic traits (Fig 5O, arrowheads), but not in companion mice injected with vehicle (Fig 5G). In addition, large osteochondromas formed along the growth plates of mutant long bones (Fig 5P, arrowheads) but not controls (Fig 5H), demonstrating that the *Ext1*^*f/f*^*;Agr-CreER* model mimics the stereotypic postnatal multiple osteochondroma phenotype of HME.

We found previously that the onset of osteochondroma formation in conditional Ext1-deficient mice is accompanied by ectopic expression of phosphorylated SMAD1/5/8 proteins in perichondrium that mediate canonical BMP signaling and are pro-chondrogenic [4, 27]. To obtain further insights, we analyzed whether there was a reciprocal loss of signaling proteins that are anti-chondrogenic. Thus, we examined pERK1/2 that mediate fibroblast growth factor (FGF) signaling known to be strongly anti-chondrogenic [54]. Indeed, we found that there was a marked decrease in immunodetectable pERK1/2 levels along the mutant synchondroses of tamoxifen-treated mice at 1 and 2 weeks from injection during the early stages of osteochondroma development, while staining was clear and abundant along the synchondroses of companion vehicle-treated mice (S1 Fig). Similar data were obtained in long bones (S1 Fig).

### Osteochondroma formation is inhibited by treatment with a BMP signaling antagonist

**Fig 6:**
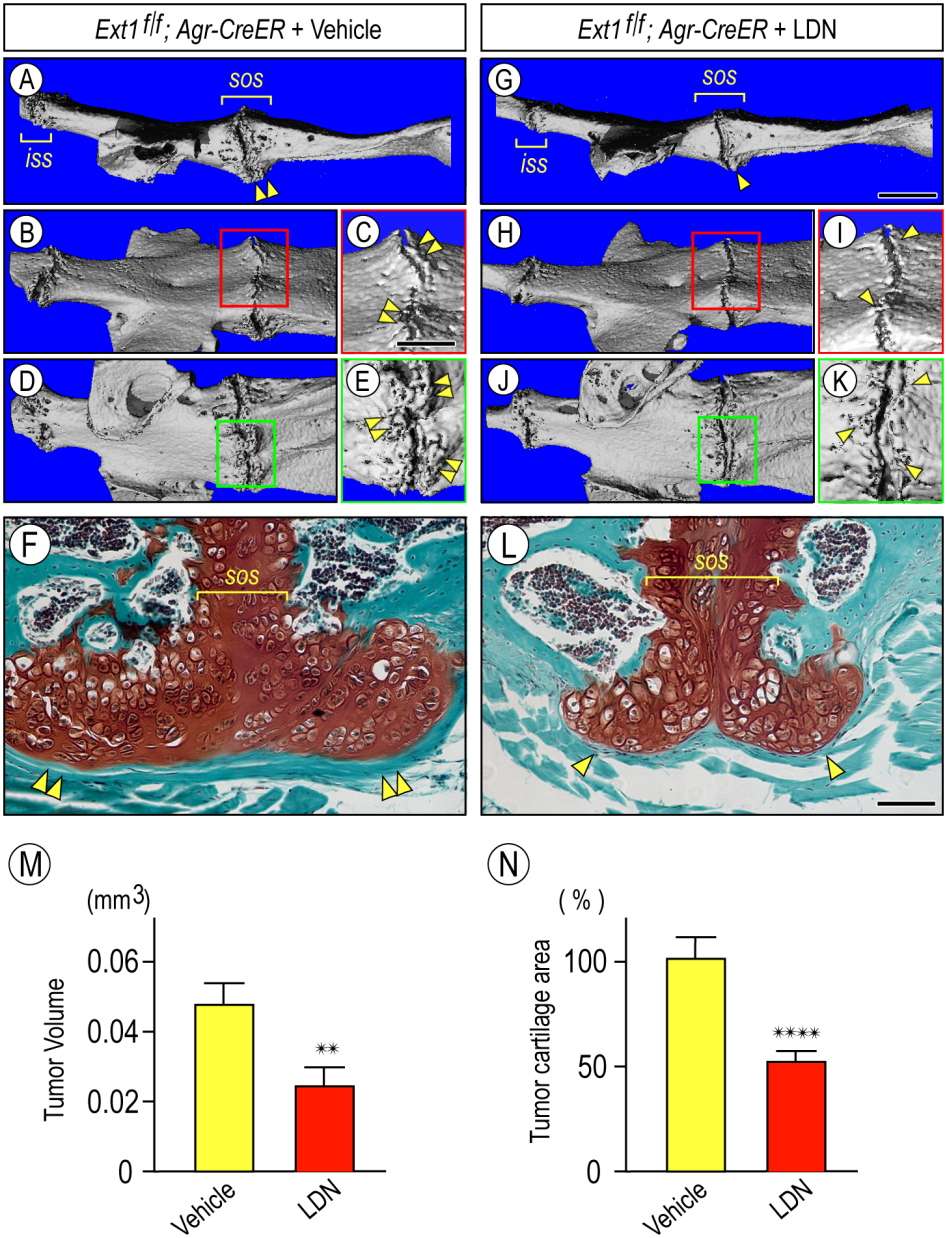
Osteochondroma development in juvenile mice is inhibited by systemic treatment with BMP signaling antagonist LDN-193189. (**A-E**) Lateral and bird’s eye μCT images of the cranial base from mutant *Ext1*^*f/f*^*;Agr-CreER* mice sacrificed 6 weeks from tamoxifen injection that were administered vehicle daily throughout the treatment period. Note the presence of multiple osteochondromas near the intrasphenoidal (*iss*) and spheno-occipital (*sos*) synchondroses highlighted by double arrowheads in A, C and E. Squared areas in B and D are shown at higher magnification in C and E. (**F**) Representative histochemical image from a serial section throughout the cranial base osteochondromas from above mutant mice. Staining with safranin O and fast green reveals the conspicuous cartilaginous portion of the tumor and presence of a thick perichondrium surrounding its distal end (double arrowheads). (**G-K**) Lateral and bird’s eye μCT images of the cranial base from mutant *Ext1*^*f/f*^*;Agr-CreER* mice sacrificed 6 weeks after tamoxifen injection that were administered LDN-193189 daily throughout that period. Note the significant and obvious reduction in osteochondroma size (arrowheads) that is best appreciable at higher magnification of squared areas in H and J shown in I and K. (**L**) Representative histochemical image from a serial section throughout the cranial base osteochondromas from above mutant LDN-treated mice. Note the reduction in the cartilaginous tumors (arrowheads). (**M** and **N**) Histograms of average bone tumor volume and cartilage tumor volume, respectively, in vehicle-treated and LDN-treated mice. Bar in (G) for A, B, D, G, H and J, 1.2 mm; bar in (C) for C, E, I and K, 0.5 mm; bar in (L) for F and L, 50 μm.

**Fig 7:**
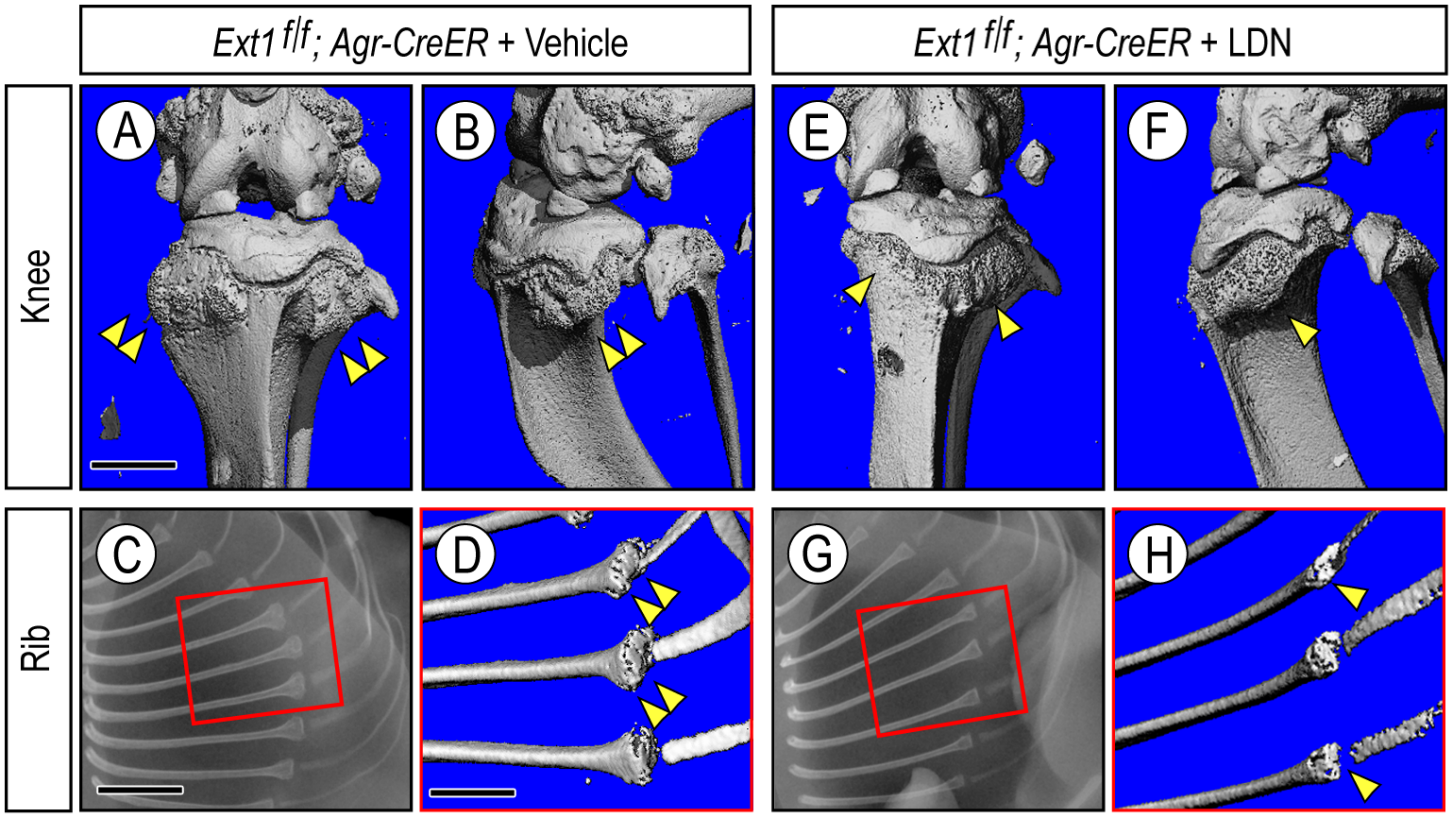
Osteochondroma development in long bones and ribs in juvenile mice is inhibited by systemic LDN-193189 treatment. **(A to D)** μCT images (A, B and D) and X-ray image (C) of knee and ribs from mutant *Ext1*^*f/f*^*;Agr-CreER* mice receiving vehicle treatment and showing large osteochondromas (double arrowheads). (**E to H**) Analogous images from companion mutant mice receiving LDN treatment for 6 weeks. Note the appreciable reduction in osteochondorma size in both knee and ribs (arrowhead). Bar in (A) for A, B, E and F, 5 mm; bar in (C) for C and G, 3 mm; and bar in (D) for D and H, 1.5 mm.

At present, there is no drug-based treatment by which osteochondroma formation could be inhibited or prevented during HME [27]. Osteochondroma development starts with chondrogenesis and cartilage formation and it is thus conceivable that drugs able to block those processes should inhibit or prevent it. Given that ectopic BMP signaling precedes onset of osteochondroma formation in HME mouse models [4] and this pathway is pro-chondrogenic [32, 55], it likely has a causative role in osteochondroma development. In related studies, others showed that small molecule antagonists of BMP signaling [56, 57] inhibited ectopic cartilage and bone formation in mouse models of the severe pediatric disorder Fibrodysplasia Ossificans Progressiva (FOP) [58] that is caused by activating mutations in the type I BMP receptor ACVR1/ALK2 [59]. To test whether BMP signaling could be a therapeutic target in HME, male and female *Ext1*^*f/f*^*;Agr-CreER* mice at 5 weeks of age were injected with tamoxifen once, randomized and then treated with LDN-193189 at 3 mg/kg/day or vehicle by daily I.P. administration, starting one day after tamoxifen injection and for a total of 6 weeks. Drug dose and regimens were similar to those successfully employed in the previous mouse FOP study [58]. To globally assess the effects of drug treatment on osteochondroma formation, we collected the skulls, rib cages and limbs from LDN-193189- and vehicle-treated mice and subjected them to μCT. In vehicle-treated mutant mice at the 6-week time point, the cranial base displayed the expected large osteochondromas on both the cranial and nasal sides (Fig 6A, 6B and 6D) best appreciable at higher magnification (Fig 6C and 6E, arrrowheads). Very interestingly, LDN-193189 treatment had effectively reduced osteochondroma development and growth in mutant mice (Fig 6G, 6H and 6J), more evident at higher magnification (Fig 6I and 6K, arrowheads). Bone volume measurement on multiple specimens from independent experiments showed that the reduction amounted to about 65% (Fig 6M, p < 0.05). Following μCT analysis, specimens were decalcified and processed for serial section, staining with safranin O and subjected to cartilage tumor volume reconstruction and quantification. Clearly, LDN-193189 treatment had markedly reduced the growth of the cartilaginous portion of the osteochondromas (Fig 6L) compared to untreated animals (Fig 6F), amounting to a decrease of about 50% (Fig 6N, p < 0.0001). Similar decreases in osteochondroma formation and size were observed in long bones and ribs of the same LDN-193189-treated mutant mice (Fig 7E–7H, arrowheads) versus vehicle-treated mutants (Fig 7A–7D, double arrowheads). There appeared to be no major side effects of drug treatment based on careful monitoring of daily mouse behavior and appearance, except for an approximately 10% decrease in average body weight (S2 Fig). We measured the lengths of femurs and tibias in LDN-193189-treated and vehicle-treated mice and found that there was no appreciable difference (S3 Fig), suggesting that the approx. 10% decrease in body weight reflected a generalized response to treatment. Similar overall observations were obtained in 4 independent experiments, and all data were used to calculate statistical differences above.

### Anti-chondrogenic action of LDN-193189 involves distinct changes in protein signaling and gene expression

**Fig 8:**
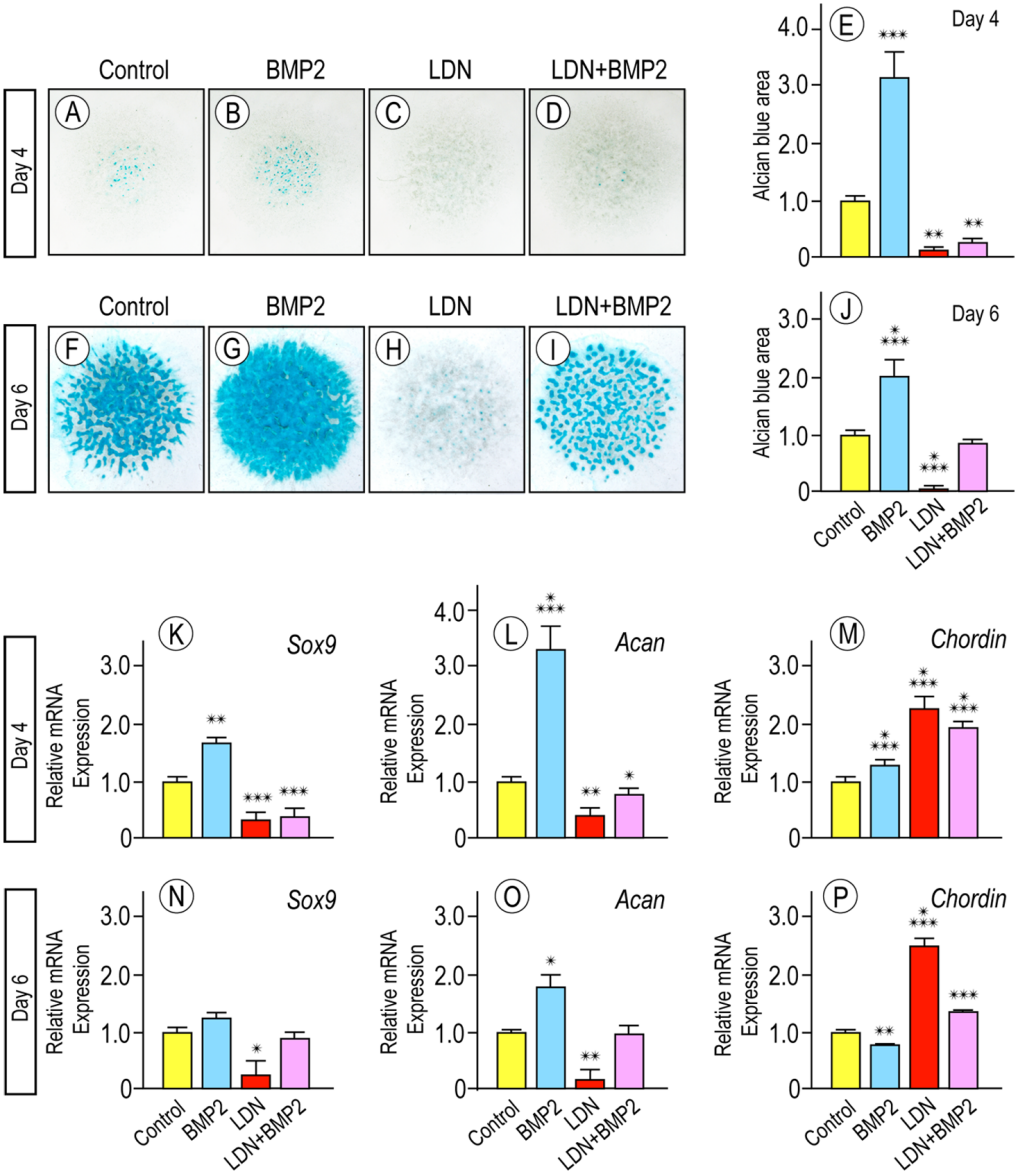
LDN-193189 is a strong and direct inhibitor of chondrogenesis. (**A-J**) Alcian blue staining and optical quantification of mouse embryo limb bud cell micromass cultures on day 4 and 6 reared in control medium (A and F) or medium containing rhBMP2 (B and G), LDN (C and H) or rhBMP2 plus LDN (D and I). Note the drastic inhibition of chondrogenesis by LDN treatment at both day 4 and 6 (C and H) and partial but still major inhibition in co-treated cultures (D and I). Quantification of alcian blue staining confirms visual data and establish statistical significance (E and J). (** p < 0.005; *** p < 0.001). (**K-P**) Histograms of qPCR data showing that LDN treatment caused significant decreases in master cartilage genes Sox9 and aggrecan (Acan) on both day 4 and 6 of culture compared to levels in control or rhBMP2-stimulated cultures, while concurrently increasing the gene expression levels of the endogenous BMP antagonist Chordin. (* p < 0.05; ** p < 0.01; *** p < 0.001).

By being an antagonist of canonical BMP signaling, it is likely that LDN-193189 was able to counter osteochondroma formation by inhibiting chondrogenesis, but this has not been tested directly. Thus, we used an established chondrogenic assay in which preskeletal mesenchymal cells isolated from E11.5 mouse embryo limb buds are seeded in micromass cultures [60]. The cells proliferate and maintain a pre-chondrogenic character in the first 2 days or so of culture and then form condensations, differentiate and produce numerous cartilage nodules by day 5 or 6. Accordingly, starting on day 1, cultures were treated with 100 nM LDN-193189, were given fresh drug every other day, and were harvested on days 4 and 6 and stained with alcian blue to reveal cartilage nodules. The LDN-193189 dose used here was selected from several tested for effectiveness and low side effects (S4 Fig). For comparison, companion cultures were treated with rhBMP2 (100 ng/ml), rhBMP2 plus LDN-193189, or vehicle and were maintained, harvested and analyzed in the same manner. As expected, control vehicle-treated cultures displayed several alcian blue-positive cartilage nodules by day 4 (Fig 8A) and many more by day 6 (Fig 8F). Companion cultures treated with rhBMP2 displayed even more (Fig 8B and 8G). However, there were very few if any cartilage nodules in companion cultures treated with LDN-193189 (Fig 8C and 8H) and appreciable reduction in nodule formation in cultures treated with rhBMP2 plus LDN-193189 (Fig 8D and 8I), indicating that the drug had significantly countered both basal and rhBMP2-stimulated chondrogenesis (Fig 8E and 8J). Gene expression analysis verified these observations and showed that LDN-193189 treatment inhibited both basal and rhBMP2-stimulated expression of chondrogenic master gene *Sox9* and cartilage matrix marker *Aggrecan* on day 4 (Fig 8K and 8L) and basal expression at day 6 (Fig 8N and 8O).

**Fig 9:**
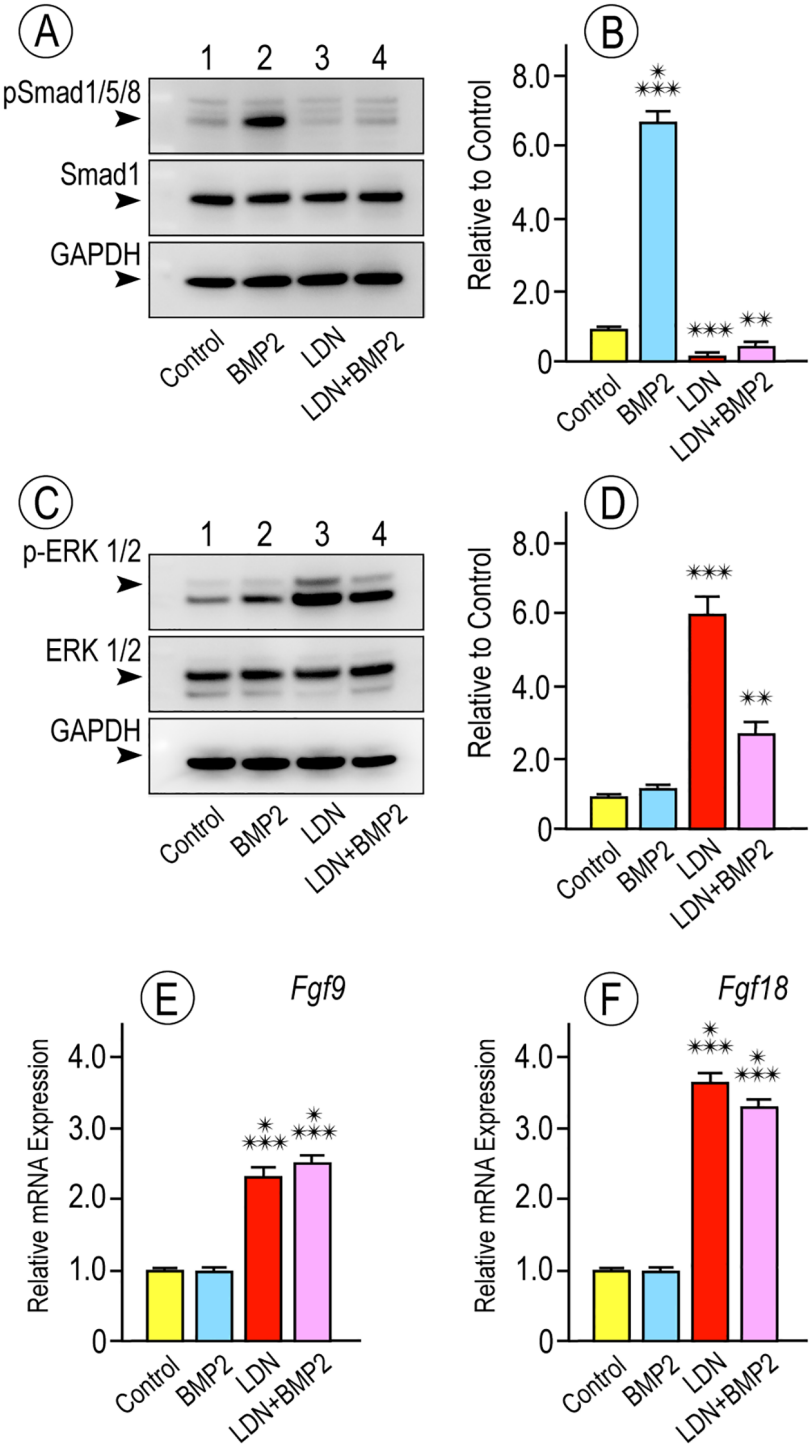
LDN-193189 differentially alters pro- and anti-chondrogenic signaling pathways and factors. (**A-D**) Immunoblot images and quantification showing that LDN treatment of micromass cultures significantly reduced levels of pro-chondrogenic pSMAD1/5/8 proteins while concurrently increasing the levels of anti-chondrogenic pERK1/2 proteins relative to controls (** p < 0.01; *** p < 0.001). (**E-F**) Histograms of qPCR data showing that LDN treatment caused a marked increase in gene expression of *Fgf9* and *Fgf18*, genes normally expressed in perichondrium that may be needed to normally maintain its fibroblastic phenotype.

Previous in vivo and in vitro studies showed that the regulation of chondrogenesis involves differential modulation of pathways that promote it -including BMP signaling- and limit it, including pERK1/2 and fibroblast growth factor (FGF) signaling [32, 33, 54, 61, 62]. To determine whether LDN-193189 affected such distinct pathways in opposite manners during the early cell commitment phases of chondrogenesis, freshly-plated micromass cultures were treated with LDN-193189, rhBMP2 or both on day 1, were given fresh drugs on day 2 for 1 to 2 hrs, and were then processed for immunoblot analysis and quantification of pSMAD1/5/8 and pERK1/2 levels. While rhBMP2 treatment increased pSMAD1/5/8 levels as expected (Fig 9A, lane 2, and Fig 9B), LDN-193189 treatment significantly reduced both basal and rhBMP2-stimulated pSMAD1/5/8 levels (Fig 9A, lanes 3 and 4, and Fig 9B). But surprisingly, LDN-193189 treatment significantly increased pERK1/2 levels, even in cultures co-treated with rhBMP2 (Fig 9C, lanes 3–4, and Fig 9D) compared to respective controls (Fig 9C, lanes 1–2). Whole cell RNAs isolated from similar cultures were processed for quantitative PCR to analyze expression of endogenous anti-chondrogenic factors. LDN-193189 treatment greatly stimulated expression of *Fgf9* and *Fgf18* (Fig 9E and 9F) that are normally expressed in perichondrium [63–65] and may be needed to maintain its fibroblastic character and prevent chondrogenesis. LDN-193189 treatment also stimulated the expression of the endogenous BMP antagonist *Chordin* by 2 to 3 fold over controls (Fig 8M and 8P).

## Discussion

The results of our study provide novel evidence that the cranial base can be affected in HME patients and when it is, exhibits defects ranging from small irregularities in clivus shape and bone contour to the presence of large osteochondroma-like outgrowths. The study provides equally compelling evidence that this phenotype is recapitulated in the cranial base of our HME model mutant mice and that osteochondroma development can be inhibited by systemic treatment with a BMP signaling antagonist, a novel contribution to the field of HME research and therapeutics. Because the mouse models are far more aggressive than the human disease given that both *Ext1* alleles are concurrently deleted in the targeted cell populations, the effectiveness of drug treatment in such models suggests that a similar pharmacologic intervention could be equally or even more effective in HME patients. Our study also provides important insights into how LDN-193189 exerts its anti-osteochondroma effects, namely by an inverse modulation of important signaling pathways likely converging to suppress ectopic chondrogenesis.

The cranial base has attracted much attention and research efforts over recent years because of its complex and still not fully understood embryology, its important role as a supporting and finely sculpted platform for the developing brain, pituitary, eyes and auditory organs as well as its involvement in congenital disorders [34, 40, 66–68]. As indicated above, much of the cranial base is endochondral and originates from the prechordal, hypophyseal and parachordal cartilaginous plates, three pairs of precursor structures that form between the first and second months of human embryogenesis [34, 35]. The parachordal plates flank the most cephalic portion of the notochord. The three pairs of plates eventually fuse to form an uninterrupted cartilaginous structure that spans the region from the interorbital junction to the occipital area. With further time, primary ossification centers emerge first posteriorly and then anteriorly, and this results in the emergence and establishment of the various intervening synchondroses. In turn, the synchondroses sustain further growth, elongation and ossification of each cranial base skeletal element and then involute and close in early childhood when cranial growth is completed [36, 69]. As a reflection of its anatomical position, the clivus largely originates from the parachordal plates and its growth is sustained by the spheno-occipital synchondrosis. It is thus possible that the defects observed in the clivus of HME patients originate from deranged function by the spheno-occipital synchondrosis in early childhood when the growth plates are very active, and the defects may have worsened over age. This interpretation fits well with our mouse data showing that incipient osteochondromas were already visible by about 2 weeks after tamoxifen injection along the cranial base synchondroses of mutant mice and became conspicuous and enlarged over time. It is important to note that the cranial base is a component of the chondrocranium that includes the cartilaginous elements around the eyes, otic pits and nasal pits as well as the exoccipital bone posterior to the foramen magnum that derives from segmented paraxial mesoderm (occipital somites) [34, 35]. We may surmise then that the deficiency in HS and derangement of growth plate function in HME patients could affect such cartilage-derived structures as well, as our patient data suggest (Fig 1). By the same token, those changes could affect other endochondral cranial base elements such as the sphenoid or ethmoid bones, suggested by our finding that osteochondromas form along the intrasphenoidal synchondrosis in mutant mice (Fig 3). Given that the sphenoid contains the *sella turcica* where the pituitary resides, changes in developmental, morphological and/or structural features of the sphenoid could possibly affect this vital gland. Testing of these possibilities and speculations will require dedicated, systematic and prospective analyses of the entire skull of HME patients over disease time, currently not prescribed but certainly to be considered in view of our data here.

Our observed defects in the clivus raise pertinent questions regarding correlations with other tumors described in that skeletal element as well as possible health consequences. Notably, the clivus is a prominent site for the development of chordomas [70–72]. These low-grade malignancies usually exhibit low metastatic tendency and are distinguished in conventional, chondroid and dedifferentiated subtypes depending on their histopathology. These tumors are associated with the clivus, but are not an integral and continuous part of it, unlike the large osteochondroma-like outgrowths described here that appear to be an uninterrupted extension of the clivus itself. The chordomas take their name by the widely held notion [70–72] that they may derive from remnants of the cephalic notochord located between the two parachordal cartilaginous plates from which the clivus itself derives in the embryo [34, 35]. While there is no direct evidence that chordomas originate from notochordal cells, what has been clear is that these tumors are associated with, and may directly cause, a number of health problems that include: visual deterioration, oculomotor nerve palsy, dislopia, brainstem compression and severe headaches [70–72]. These clinical findings suggest that the defects we observe in the cranial base of HME patients could have clinical consequences. HME is mostly known for the axial and appendicular skeletal problems caused by osteochondroma formation and their possible transformation into malignant and life-threatening chondrosarcomas. However, HME patients can experience other problems that include chronic pain, social difficulties, and daily and occupational life impairments [8, 73]. It is unclear at this point whether the defects in the clivus or other skull elements could have health consequences, for example due to impingement of the pons or local arteries by a large protruding osteochondroma. As above, direct clinical studies will be needed, but if evidence were to emerge, the findings could lead to new standard of care protocols for this patient population.

A lingering and frustrating issue in HME is that beside surgery to remove the most symptomatic and accessible osteochondromas, this disease does not have yet alternative drug-based remedies [27]. Possible therapeutic targets have emerged from previous studies, one example being heparanase that we and others found to be up-regulated in human and mouse osteochondromas [74, 75], but no study has tested efficacy. Thus, our current data are a wholly novel contribution to the field and show for the first time that osteochodroma formation can be inhibited by systemic treatment with the BMP signaling antagonist LDN-193189 in cranial base, long bones and ribs, regardless of incidence at each site and size of ectopic tissue mass. Our in vitro data with chondroprogenitor cells in micromass cultures suggest that LDN-193189 likely inhibited tumor formation by reducing chondrogenic mechanisms including *Sox9* expression and pSMAD1/5/8 signaling [32, 55] while enhancing anti-chondrogenic pathways including pERK1/2 signaling and *Fgf* gene expression [54, 61, 62]. This comprehensive action, combined with the modulation of *Chordin* expression, could certainly account for the powerful effects of LDN-193189. The anti-chondrogenic potency of this drug is actually not wholly surprising since it was documented in the mouse studies on FOP, an aggressive condition involving extraskeletal cartilage and endochondral bone formation and accumulation [58]. Several important questions remain to be answered. We do not know whether LDN-193189 treatment would still be effective if it were initiated after osteochondroma onset and what the window of opportunity for this drug may be. In a reciprocal manner, LDN-193189 could be more effective if given prior to osteochondroma initiation or even chronically and if it were to be tested in less aggressive HME mouse models, such as single *Ext1*^*+/-*^ or double heterozygous *Ext1*^*+/-*^*;Ext2*^*+/-*^ mice [24]. In these two models, osteochondroma formation is sporadic and occurs at different times and sites in manners reminiscent of tumor formation in HME patients. These models would thus be ideal to test the efficacy of chronic treatments and also whether certain anatomical sites are preferentially suitable for osteochondroma formation, a possible reflection of special microenvironmental niches or particular progenitor cell populations. These and other experiments could thus produce important insights into drug potency and malleability, osteochondroma developmental steps and hierarchy and drug sensitivity of regulatory mechanisms. On the other hand, it needs to be pointed out that powerful drugs do not always translate into clinically relevant remedies since they could have side effects. LDN-193189 targets pSMAD1/5/8 signaling, but can also affect other pathways [76]. Fortunately, derivatives of LDN-193189 have been designed and found to have more specific effects [56, 77], possibly offering safer therapeutic options. Despite its limitations, the present study provides much needed proof-of-principle evidence that osteochondroma formation can be inhibited by drug treatment in HME mouse models. Our study thus represents a major step toward the development of an effective drug-based treatment against this serious and often debilitating disease.

## Materials and methods

### Retrospective analysis of patient MRI and CT scans

These studies were reviewed and approved by the IRB (protocol no. 00072788, principal investigator KBJ) at the University of Utah and were designed as an exempt study. The cephalad extent of the whole spine three-dimensional imaging scans from 50 consecutive pediatric HME patients at the University of Utah were re-examined by two physicians independently and assessed by standard radiological criteria for abnormalities in the foramen magnum, occiput and clivus. Aberrations from normal were categorized as either irregularities of osteochondromas, the latter category requiring the presence of clear cortical and medullary continuity with the underlying host bone and the surface excrescence. Data were subjected to 2-tailed t-test for statistical significance.

### Ethics statement regarding mouse studies

All experiments involving wild type and transgenic mice were reviewed by the IACUC at The Children’s Hospital of Philadelphia (protocol no. IAC 14–000952, principal investigator MP). Animals were handled, treated and cared for according to the approved protocols and procedures.

### Transgenic mouse lines, husbandry and drug treatment

LoxP-modified *Ext1*^*f/f*^ mice described previously [20] were mated with *Col2a1-CreERT* (abbreviated to *Col2-CreER*) transgenic mice expressing *Cre* recombinase linked to modified estrogen ligand binding domain under the control of *collagen2a1* enhancer sequences [50] to generate compound *Col2CreER;Ext1*^*f/f*^ mice. Companion control mice included *Col2CreER*, *Col2CreER;Ext1*^*f/+*^ or *Ext1*^*f/f*^ mice. Mice were used for phenotypic analyses of osteochondroma formation and growth in cranial base and other sites as in previous related studies [20, 22, 49]. Control and compound transgenic mice at P7 or P10 were given a single intraperitoneal injection of tamoxifen (1 mg per 13 grs body weight); stock tamoxifen solution was 20 mg/ml in ethanol:corn oil mixture at 1:4 ratio. When indicated, companions received a similar volume of ethanol:corn oil vehicle. Mice were sacrificed at indicated time points, and body parts and tissues were processed for imaging and other procedures as detailed below. For experiments at juvenile stages, we used *Ext1*^*f/f*^ mice [49] mated with *Aggrecan-CreERT2* (*Agr-CreER*) mice [53] to generate compound *Agr-CreER;Ext1*^*f/f*^ mice and appropriate controls. Mice at P28 or P35 were then treated with tamoxifen or vehicle as above. To monitor topography of *CreER* action, the *Agr-CreER* mice were mated with *R26-tdTomato* reporter mice (Jackson Labs). Compound *Agr-CreER*;*R26-tdTomato* mice were injected with tamoxifen or vehicle at P21, P28 or P35, and limb and craniofacial specimens were harvested 2 to 4 days later and processed for histological and fluorescence analysis of reporter activity as described [78]. Labeling and analysis of proliferative cells by EdU incorporation were carried out as described [79].

For experiments involving the BMP signaling inhibitor LDN-193189, the drug was dissolved in distilled water at 1 mg/ml stock solution [58]. Aliquots were prepared and stored at -80°C. On the day of treatment, an aliquot was thawed and used only once to treat mice at 3 mg/kg dose by IP injection once a day for a total of 6 weeks. Companion controls were injected with vehicle (water). Treatment started one day after tamoxifen injection. Each group consisted of 3–4 vehicle-treated and 3–4 drug-treated mice. We carried out a total of five independent experiments, and data were used to calculate averages and statistical significance.

### Histological, histochemical, x-ray and μCT analyses

Indicated body parts and samples were fixed overnight in 4% paraformaldehyde, washed with 1x PBS for 3 times and stored in PBS or ethanol at 4°C. Whole cranial bases were scanned for μCT in coronal and sagittal view using a Viva CT 40 scanner (Scanco Medical AG, Switzeland) and analyzed using μCT v6.0 vivaCT software as we described previously [80]. Serial 10.5 μm 2D and 3D images were acquired at 55 kVp energy, 145 μA intensity and integration time of 200 msec. Raw μCT data were compiled into 2D gray scale images. Cranial base in coronal scans was contoured, and binary images were generated using a threshold of 330. Virtual 3D models were then constructed and analyzed for morphological abnormalities. For determination of osteochondroma volume in control versus LDN-193189-treated mice, we used sagittal scans. For this purpose, the spheno-occipital synchondroses were contoured to define their entire width, usually requiring 204 to 228 slices in the control groups and 203 to 219 slices in the drug-treated groups. Because osteochondromas were more prominent on the nasal side, tumor volumes were measured in that region by including a central slide through tumor midpoint and sufficient neighboring slices on left and right directions (usually 12 each) to encompass the tumor. Bone volumes were measured using μCT v6.0 vivaCT software, using a threshold of 330. Acquired values were then tested for significance using an unpaired non-parametric t-test and GraphPad Prism 6.0 (GraphPad Software, Inc., La Jolla, CA, USA). Significance level was set at 1% (*p* < 0.01). For analyses of ribs and knee, we used a Scanco μCT 35. Samples were scanned at a resolution of 21 μm, but all the other parameters were the same. Thresholds were set at 200 and 260, respectively. When indicated, specimens were examined by x-ray, using a Bioptics piXarray 100 (Kenmore, WA) set at automatic exposure control mode [22]. Images were used to measure the lengths of femurs and tibias from vehicle-treated and drug-treated mice in each experiment performed, and resulting data were used to calculate averages and statistical significance as indicated below.

After performing imaging procedures, cranial base, long bone and rib samples were decalcified and embedded in paraffin. Resulting serial 8 μm sections were stained with safranin-O and fast green [78]. Cumulative area of cartilage was quantified as described [81]. Briefly, images of one in every 4 consecutive sections encompassing the entire cartilaginous tumor were analyzed using ImageJ software. Following threshold adjustment, histological region of interest was set and the area of cartilage within that region in the consecutive sections was calculated. Data were expressed as cartilage area over total region area.

### In situ hybridization

Paraffin sections were processed for in situ hybridization as described [41]. Briefly, after paraffin removal and rehydration, serial tissue sections were pretreated with 10 μg/ml proteinase K for 10 min at room temperature, post-fixed in 4% paraformaldehyde, washed with PBS containing 2 mg /ml glycine, and treated with 0.25% acetic anhydride in triethanolamine buffer. Sections were hybridized with antisense or sense ^35^S-labeled riboprobes (approximately 1x10^6^ DPM/section) at 50°C for 16 hrs, coated with Kodak NTB-3 emulsion diluted 1:1 with water, and exposed for 10 to 14 days. Slides were developed with Kodak D-19 at 20°C and stained with hematoxylin. cDNA clones used as templates for probe transcription included: *collagen I* (nt. 233–634; NM_007742); *collagen II* (nt.1095-1344; X_57982), *collagen X* (nt.1302-1816; NM_009925) and *Mmp13* (nt. 11–744; NM_008607).

### Preparation, treatment and analysis of micromass cultures

Micromass cultures were prepared from E11.5 CD-1 mouse embryo limb buds as described [60]. Briefly, limb bud mesenchyme was dissociated in 0.5% trypsin-EDTA at 37°C. The dissociated cells were suspended at a concentration of 10 x 10^6^ cells/ml in DMEM containing 3% fetal bovine serum and antibiotics. Micromass cultures were initiated by spotting 15 μl of the cell suspensions (1.5 x 10^5^ cells) onto the surface of 24-well tissue culture plates. After a 2 h incubation at 37°C in a humidified CO_2_ incubator to allow for cell attachment, the cultures were given 500 μl of medium. After 24 h, medium supplemented with LDN-193189 (100 nM) (cat# SML-0559, Sigma-Aldrich), rhBMP-2 (100 ng/ml) (cat# 300-103P, Gemini Bio), or a combination of LDN-193189 (100 nM) plus rhBMP-2 (100 ng/ml) was added to the cultures. Fresh reagents (drug and/or protein) were given with medium change every other day. Equivalent amounts of vehicle (1X PBS or 4 mM HCl) were added to control cultures. Cultures were stained with Alcian blue (pH 1.0) after 4 days to monitor chondrogenic cell differentiation [4]. Images were taken with a Nikon SMZ-U microscope equipped with a SPOT insight camera (Diagnostic Instruments, Inc.) and acquired with SPOT 4.0 software. Micromass analysis was performed using ImageJ. Images were made binary under an RGB threshold and “Particle Analysis” was utilized to measure Alcian blue positive area [81].

### Gene expression analysis

Total RNA was isolated from control, LDN193189-treated, rhBMP-2-treated and LDN193189/rhBMP-2-treated micromass cultures on day 4 using TRIzol reagent (cat# 15596–026, Life Technologies) according to the manufacturer’s protocol. We determined RNA quantification by Nanodrop. One microgram total RNA was reversed transcribed using the Verso cDNA kit (cat# AB1435/A, Thermo Scientific). Quantitative real-time PCR was carried out using SYBR Green PCR Master Mix in an Applied Biosystems 7500 according to manufacturer’s protocol. *Gapdh* was used as the endogenous control and relative expression was calculated using the ΔΔCt method. Primer information can be found in Supplemental Table 1.

### Protein analysis in cell cultures and tissues

Micromass cultures were treated with vehicle, LDN193189 (100 nM), rhBMP-2 (100 ng/ml) or LDN193189 (100 nM) plus 100 ng/ml rhBMP-2) for the first two days and were then acutely treated for 1 hr with fresh factor(s) before harvest. Cultures were lysed in 1X RIPA with protease and phosphatase inhibitors, and samples were centrifuged at 13,200 rpm at 4°C and supernatants were collected. Protein concentration for each sample was determined using the MicroBCA Protein Assay Kit (cat# 23235, Thermo Scientific) according to the manufacturer’s protocol. Total cellular proteins (30 μg/lane) were electrophoresed on 4–12% NuPAGE Bis-Tris gel (cat# NP0322, Life Technologies) and transferred to PVDF membranes (cat# LC2005, Life Technologies). Membranes were blocked in 5% BSA/1X Tris Buffered Saline/Tween 20 (TBST) and incubated overnight at 4°C with one of the following antibodies: phospho-SMAD1/5/8 (pSMAD1/5/8) (1:1000; cat# 13820, Cell Signaling) or phospho-ERK1/2 (1:1000; cat# 4370, Cell Signaling). Membranes were washed in 1X TBST and incubated with anti-rabbit HRP-linked antibodies (1:2000; cat# 7074, Cell Signaling) for 1 hr at room temperature. Antigen-antibody complexes were detected with SuperSignal^®^ West Dura Extended Duration Substrate (cat# 34075, Thermo Scientific) chemiluminescent detection system. Membranes were re-blotted with SMAD1 (1:1000; cat# 9743, Cell Signaling) or ERK1/2 (1:1000; cat# 9102, Cell Signaling) antibodies for normalization. For loading control, membranes were blotted with GAPDH antibodies (1:1000; cat# 32233, Santa Cruz Biotechnology). ImageJ was used to determine band intensities.

Above antibodies to pSMAD1/5/8 and pERK1/2 were used to carry out immunohistochemical analyses in serial sections of cranial base and long bones from control and conditional *Ext1*-deficient mice, following the protocol described previously [4, 27].

### Statistical analysis

Results were analyzed using InStat 3 version 3.1a (GraphPad Software, Inc., La Jolla, CA). A one-way Analysis of Variance (ANOVA) with a Tukey-Kramer Multiple Comparison Test or Student’s t-test was used to identify the differences. Threshold for significance for all tests was set as p<0.05.

## Supporting information

S1 Table Sequences of forward and reverse primer pairs used to determine the gene expression levels of indicated genes in cultured cells. Genes are designed by their acronyms routinely used in the literature as well as by their accession number at GeneBank. Expected sizes of PCR products are specified as well.
(PDF)

S1 Fig Mutant perichondrium exhibits decreases in anti-chondrogenic proteins. (A and D) Longitudinal sections along the spheno-occipital synchondrosis from mutant *Ext1*^*f/f*^*;Agr-CreER* mice and companion *Ext1*^*f/f*^ controls sacrificed about 2 weeks after tamoxifen injection and stained with pErk1/2 antibodies. Note that positive nuclear staining is appreciable in control cells along the perichondrium (arrowheads) and within growth plate zones (A), whereas staining is much reduced in mutant (D). (**B-C** and **E-F**) Longitudinal sections of tibia from similar control and mutant mice were stained with pErk1/2 antibodies as well antibodies to Noggin, another powerful anti-chondrogenic protein expressed in perichondrium and growth plate. Note that both pErk1/2 and Noggin staining was decreased in mutant perichondrium (E-F, pc) while it was clear and well appreciable in control (B-C, arrowheads). Bar in (A) for A-F, 150 μm.
(TIF)

S2 Fig Average body weight during drug treatment. Graph shows average body weight measured in vehicle- and LDN-treated male and female mice at weekly intervals over the treatment time. Note the slight decrease in LDN-treated mice. Data are from two independent experiments involving 3–4 control and treated mice each and are presented as average values ± S.D.
(TIF)

S3 Fig Average long bone lengths after drug treatment. Graph shows average longitudinal lengths of femurs and tibias in vehicle- and LDN-treated male and female mice after 6 or 8 weeks of treatment. Note there was no appreciable difference. Data are from four independent experiments involving 3–4 control and treated mice each and are presented as average values ± S.D.
(TIF)

S4 Fig Anti-chondrogenic effects of LDN-193189 treatment. (**A to E**) Images of day 6 alcian blue-stained micromass cultures that were treated with vehicle (A) or increasing concentrations of LDN-193189 (B-E). Note the dose-dependent decrease in cartilage nodule formation. (**F**) Quantification of alcian blue-positive areas in control versus drug-treated cultures. (**** p < 0.0001). Data are averages from six independent experiments.
(TIF)
