# Supplementary material for: Unsuspected osteochondroma-like outgrowths in the cranial base of Hereditary Multiple Exostoses patients and modeling and treatment with a BMP antagonist in mice
Source: PLoS Genet. 2017 Apr 26;13(4):e1006742. doi: 10.1371/journal.pgen.1006742 (PMC5425227; doi:10.1371/journal.pgen.1006742)
Supplement: S1 Table — Genes are designed by their acronyms routinely used in the literature as well as by their accession number at GeneBank. Expected sizes of PCR products are specified as well. (PDF) [file pgen.1006742.s001.pdf]

**Supplemental Table 1**. Primers used to determine expression of chondrogenic and anti-chondrogenic genes

| Gene | Accession No. | Forward Sequence (5'-3') | Reverse Sequence (5'-3') | Size (bp) |
|---|---|---|---|---|
| ***Acan*** | NM_007424 | ggagcgagtccaactcttca | cgctcagtgagttgtcatgg | 120 |
| ***Chrd*** | NM_009893.2 | attacctgcagatgtggggc | gggttctagtctcagaggacctt | 136 |
| ***Fgf9*** | NM_013518.4 | gcagtcacggacttggatca | aattccagaatgccgaagcg | 143 |
| ***Fgf18*** | NM_008005.2 | tggggaagcctgatggtact | cccttggggtaacgcttcat | 193 |
| ***Sox9*** | NM_0011448 | gagctcagcaagactctggg | cggggctggtacttgtaatc | 131 |
| ***Gapdh*** | NM_008084 | atcttgggctacactgagga | caggaaatgagcttgacaaagt | 122 |
