# Supplementary figures and images for: Unsuspected osteochondroma-like outgrowths in the cranial base of Hereditary Multiple Exostoses patients and modeling and treatment with a BMP antagonist in mice
Source: PLoS Genet. 2017 Apr 26;13(4):e1006742. doi: 10.1371/journal.pgen.1006742 (PMC5425227; doi:10.1371/journal.pgen.1006742)

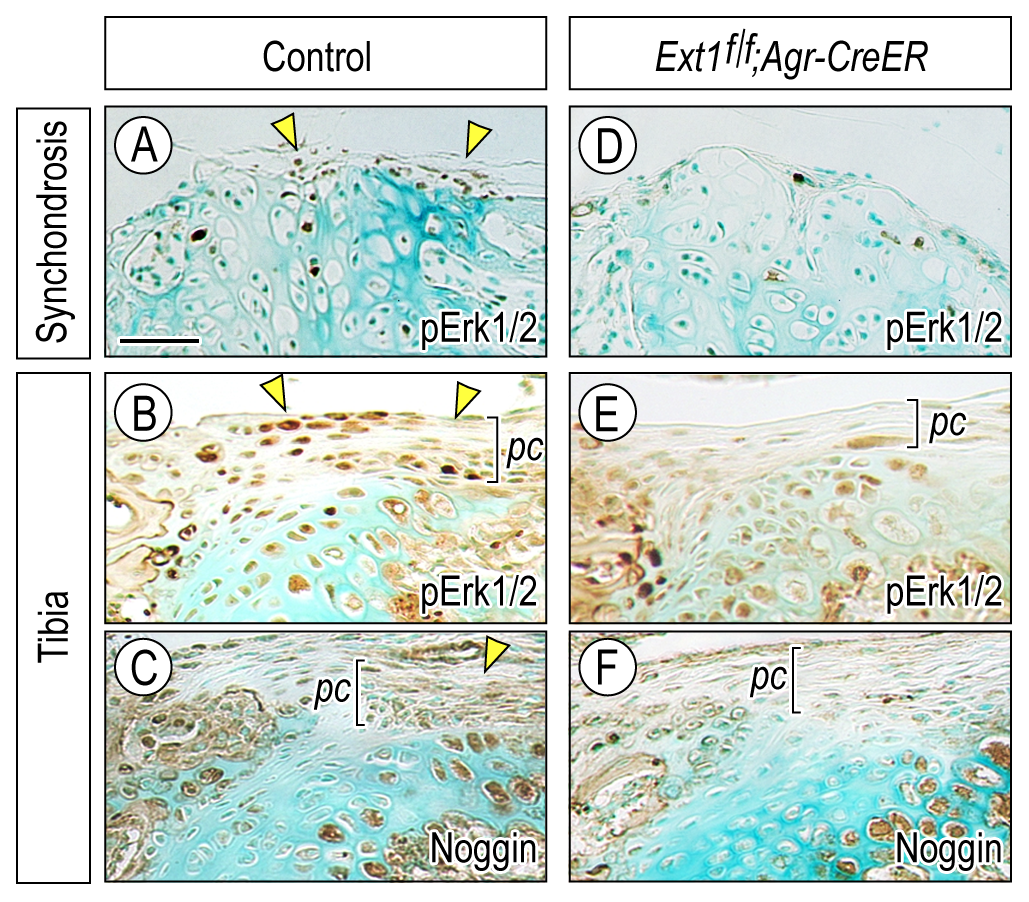

Supplement: S1 Fig — (A and D) Longitudinal sections along the spheno-occipital synchondrosis from mutant Ext1f/f;Agr-CreER mice and companion Ext1f/f controls sacrificed about 2 weeks after tamoxifen injection and stained with pErk1/2 antibodies. Note that positive nuclear staining is appreciable in control cells along the perichondrium (arrowheads) and within growth plate zones (A), whereas staining is much reduced in mutant (D). (B-C and E-F) Longitudinal sections of tibia from similar control and mutant mice were stained with pErk1/2 antibodies as well antibodies to Noggin, another powerful anti-chondrogenic protein expressed in perichondrium and growth plate. Note that both pErk1/2 and Noggin staining was decreased in mutant perichondrium (E-F, pc) while it was clear and well appreciable in control (B-C, arrowheads). Bar in (A) for A-F, 150 μm. (TIF) [file pgen.1006742.s002.tif]

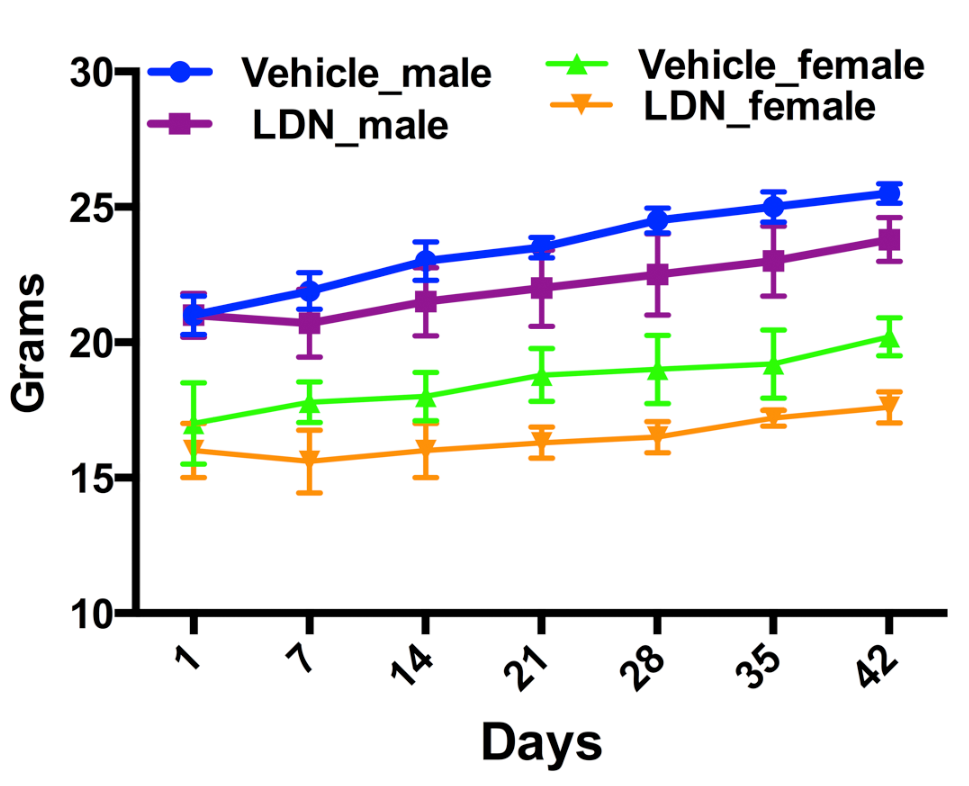

Supplement: S2 Fig — Graph shows average body weight measured in vehicle- and LDN-treated male and female mice at weekly intervals over the treatment time. Note the slight decrease in LDN-treated mice. Data are from two independent experiments involving 3–4 control and treated mice each and are presented as average values ± S.D. (TIF) [file pgen.1006742.s003.tif]

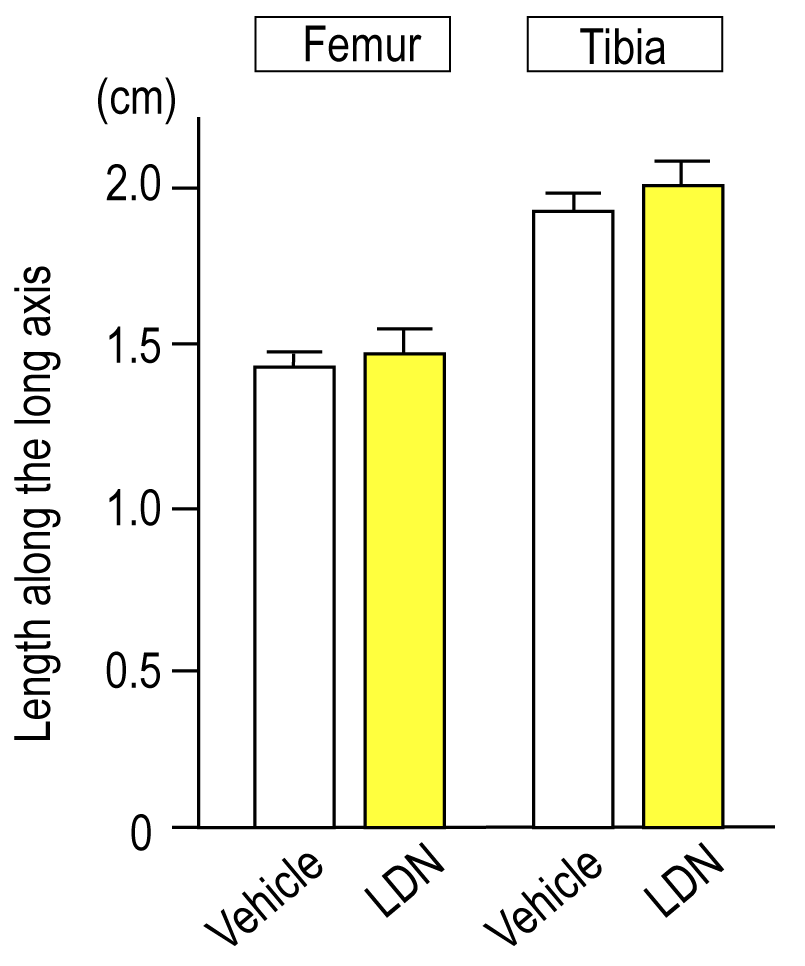

Supplement: S3 Fig — Graph shows average longitudinal lengths of femurs and tibias in vehicle- and LDN-treated male and female mice after 6 or 8 weeks of treatment. Note there was no appreciable difference. Data are from four independent experiments involving 3–4 control and treated mice each and are presented as average values ± S.D. (TIF) [file pgen.1006742.s004.tif]

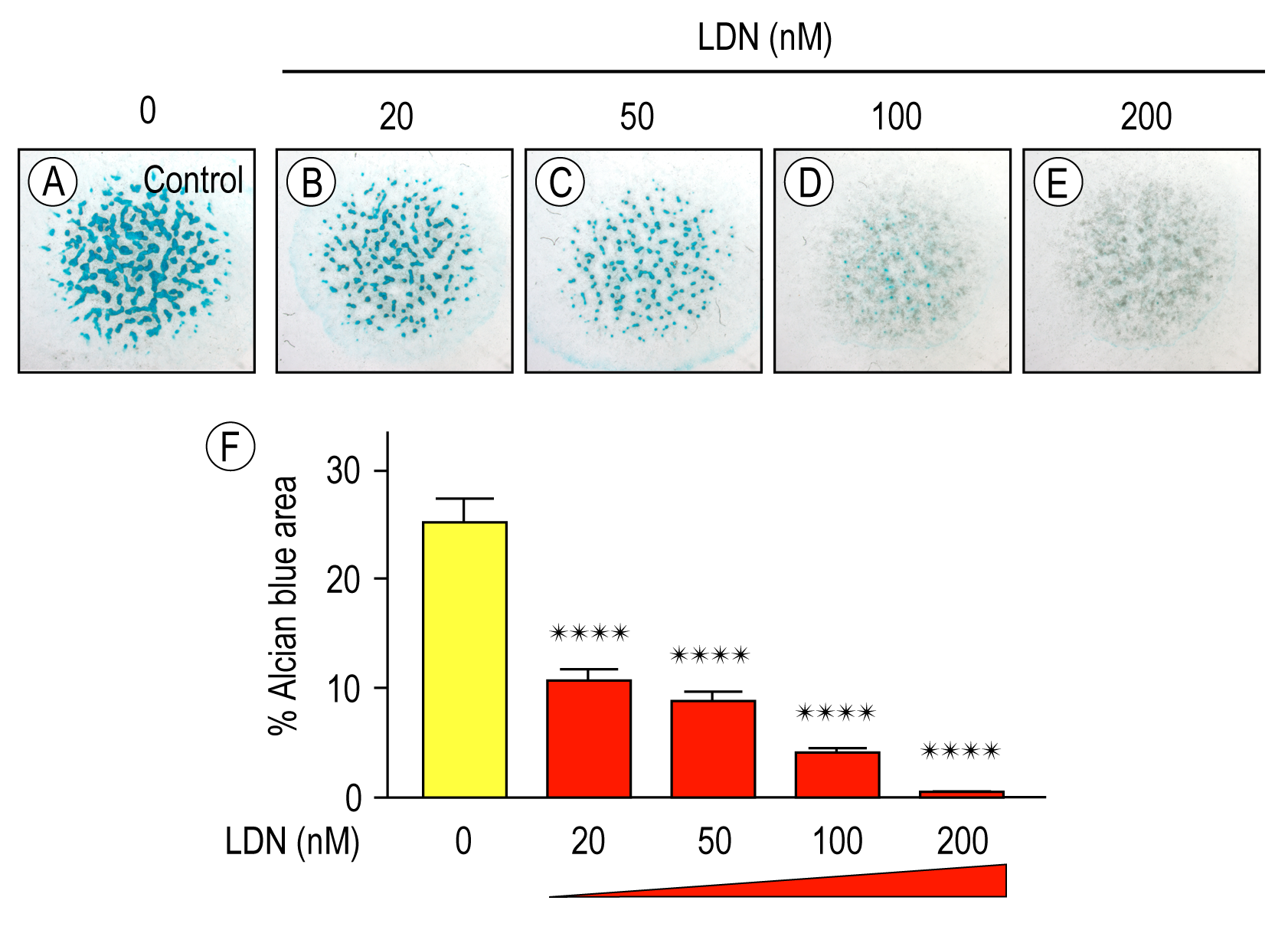

Supplement: S4 Fig — (A to E) Images of day 6 alcian blue-stained micromass cultures that were treated with vehicle (A) or increasing concentrations of LDN-193189 (B-E). Note the dose-dependent decrease in cartilage nodule formation. (F) Quantification of alcian blue-positive areas in control versus drug-treated cultures. (**** p < 0.0001). Data are averages from six independent experiments. (TIF) [file pgen.1006742.s005.tif]
